# Metabolite Changes in the Aqueous Humor of Patients With Retinal Vein Occlusion Macular Edema: A Metabolomics Analysis

**DOI:** 10.3389/fcell.2021.762500

**Published:** 2021-12-21

**Authors:** Xiaojing Xiong, Xu Chen, Huafeng Ma, Zheng Zheng, Yazhu Yang, Zhu Chen, Zixi Zhou, Jiaxin Pu, Qingwei Chen, Minming Zheng

**Affiliations:** ^1^ Department of Ophthalmology, Second Affiliated Hospital of Chongqing Medical University, Chongqing, China; ^2^ Department of general practice, Second Affiliated Hospital of Chongqing Medical University, Chongqing, China

**Keywords:** retinal vein occlusion, macular edema, macular central thickness, aqueous humor, metabolomics analysis

## Abstract

Macular edema (ME) is the main cause of visual impairment in patients with retinal vein occlusion (RVO). The degree of ME affects the prognosis of RVO patients, while it lacks objective laboratory biomarkers. We aimed to compare aqueous humor samples from 28 patients with retinal vein occlusion macular edema (RVO-ME) to 27 age- and sex-matched controls by ultra-high-performance liquid chromatography equipped with quadrupole time-of-flight mass spectrometry, so as to identify the key biomarkers and to increase the understanding of the mechanism of RVO-ME at the molecular level. Through univariate and multivariate statistical analyses, we identified 60 metabolites between RVO-ME patients and controls and 40 differential metabolites in mild RVO-ME [300 μm ≤ central retinal thickness (CRT) < 400 μm] patients compared with severe RVO-ME (CRT ≥ 400 μm). Pathway enrichment analysis showed that valine, leucine, and isoleucine biosynthesis; ascorbate and aldarate metabolism; and pantothenate and coenzyme A biosynthesis were significantly altered in RVO-ME in comparison with controls. Compared with mild RVO-ME, degradation and biosynthesis of valine, leucine, and isoleucine; histidine metabolism; beta-alanine metabolism; and pantothenate and coenzyme A biosynthesis were significantly changed in severe RVO-ME. Furthermore, the receiver operating characteristic (ROC) curve analysis revealed that adenosine, threonic acid, pyruvic acid, and pyro-L-glutaminyl-l-glutamine could differentiate RVO-ME from controls with an area under the curve (AUC) of >0.813. Urocanic acid, diethanolamine, 8-butanoylneosolaniol, niacinamide, paraldehyde, phytosphingosine, 4-aminobutyraldehyde, dihydrolipoate, and 1-(beta-D-ribofuranosyl)-1,4-dihydronicotinamide had an AUC of >0.848 for distinguishing mild RVO-ME from severe RVO-ME. Our study expanded the understanding of metabolomic changes in RVO-ME, which could help us to have a good understanding of the pathogenesis of RVO-ME.

## Introduction

Retinal vein occlusion (RVO) is the major cause of vision loss by retinal vascular diseases. Classified by the location of obstruction, RVO can be differentiated into central retinal vein occlusion (CRVO) and branch retinal vein occlusion (BRVO). Known risk factors for RVO include hypertension, atherosclerosis, hyperlipidemia, diabetes, thrombosis, and other inflammatory and myeloproliferative diseases (Petr, 2014; Balaratnasingam et al., 2016). Clinical presentations of RVO include retinal hemorrhage, tortuous retinal veins, optic nerve swelling, and macular edema (ME) (Querques et al., 2013). Among them, the most common cause of vision loss in RVO is ME. Studies have shown that central retinal thickness (CRT) was closely related to visual acuity and prognosis (Eng and Leng, 2020). Although the diagnosis of retinal vein occlusion macular edema (RVO-ME) was undoubted, the initial pathogenesis and following pathophysiology of RVO-ME remained controversial.

Abundant metabolomics studies have been carried out in animal models or humans under pathophysiological conditions to identify the most important metabolites in various ophthalmic diseases by analyzing blood or intraocular fluid samples (Deng et al., 2020). Aqueous humor (AH) provides nutrition for the surrounding avascular cornea and lens and discharges the metabolic waste from the eyes to the venous blood. The metabonomic information of AH could directly reflect the physiological state of the eyes (Haines et al., 2018). Recent studies using liquid chromatography−mass spectrometry (LC-MS) have also identified almost 250 metabolites belonging to 47 metabolic pathways in AH (Karolina et al., 2017). Additionally, wet age-related macular degeneration (Han et al., 2020), diabetic retinopathy (Pietrowska et al., 2018), severe myopia (Ji et al., 2017), primary open-angle glaucoma (Buisset et al., 2019), and primary congenital glaucoma (Breda et al., 2020) were also found to be associated with metabolomic signatures in the aqueous humor. However, AH metabolism of RVO-ME has not been reported yet.

**FIGURE 1:**
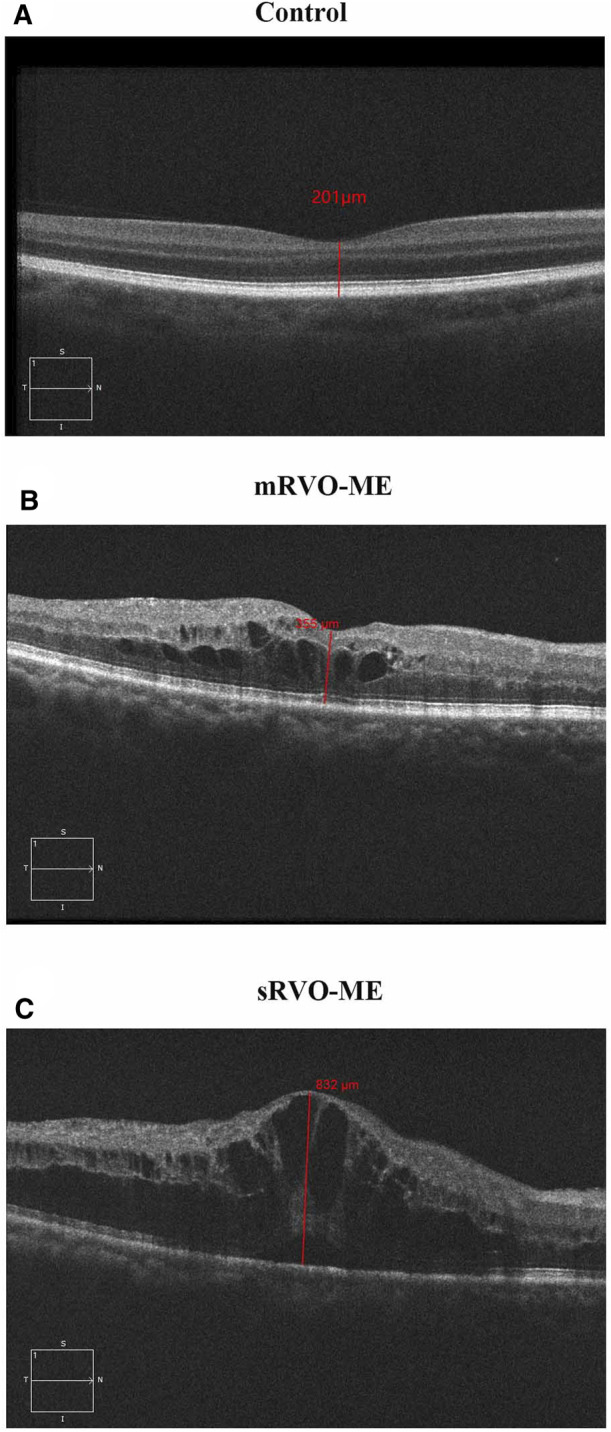
Macular central thickness in each group.

The aims of the current study were to identify differential metabolites in the RVO-ME compared with controls, to screen biomarkers from these differential metabolites, and to identify potential biomarkers that could differentiate patients between mild RVO-ME (mRVO-ME) (300 μm ≤ CRT < 400 μm) and severe RVO-ME (sRVO-ME) (CRT ≥ 400 μm) (Kim et al., 2020) (Figure 1).

## Methods

### Sample Collection

The study was conducted in accordance with the requirements of the Ethics Committee of The Second Affiliated Hospital of Chongqing Medical University, which approved the study (2020405). The study follows the principles of the Helsinki Declaration. All aqueous humor samples from patients with RVO-ME (*n* = 28) and the age- and sex-matched control group (*n* = 27) were collected from ophthalmology department of the Second Affiliated Hospital of Chongqing Medical University, from October 2020 to March 2021. All participants were informed and signed the informed consent.

Cataract grading had been assessed using the Lens Opacities Cataract Classification System III (LOCS III) (Chylack et al., 1993). LOCS III of both the RVO group and the control group were N2C2P2. The diagnosis of RVO was made using the International Classification of Diseases, Ninth Revision, Clinical Modification (ICD-9-CM). CRVO was defined as ICD-9 362.35 and BRVO as ICD-9 362.36 (Kupka, 1978). The inclusion criteria for RVO-ME were as follows: 1) age ≥18 years, 2) diagnosis within 1 year, and 3) CRT ≥ 300 μm. Exclusion criteria included the following: 1) age-related macular degeneration; 2) diabetic retinopathy; 3) previous intravitreal injection of anti-vascular endothelial growth factor or steroids; 4) previous intraocular surgery; 5) previous retinal photocoagulation; 6) glaucoma, including neovascular glaucoma; 7) iris redness and anterior chamber hemorrhage; 8) vitreous hemorrhage and other vitreoretinal disease; 9) cerebrovascular accident or myocardial infarction in the past 3 months; and 10) any kind of eye drops has been used within 3 months prior to sample collection. Samples of the control group were collected from age- and sex-matched patients who received aqueous humor samples before cataract surgery. All subjects and controls were not using hormonal medication.

### Sample Preparation

AH samples were taken by puncture after surface anesthesia and disinfection. Approximately 200 μl of aqueous humor was collected. The AH samples were immediately transferred to dust-free Eppendorf tubes, centrifuged twice at 4°C and 16,000×*g* for 15 min, and then the supernatants were collected in cryogenic vials. Finally, the supernatant was collected and quickly stored at−80°C until metabolomics analysis.

For metabolomics analysis, ultra-high-performance liquid chromatography equipped with quadrupole time-of-flight mass spectrometry (UHPLC-Q-TOF/MS) analysis has been carried out. To an EP tube, 50 μl of sample was transferred. After the addition of 200 μl of extract solution (acetonitrile/methanol = 1:1, containing isotopically labelled internal standard mixture), the samples were vortexed for 30 s, sonicated for 10 min in ice water bath, and incubated for 1 h at −40°C to precipitate proteins. Then, the samples proceeded to centrifugation at 12,000 rpm [RCF = 13,800 (×*g*), *R* = 8.6 cm] for 15 min at 4°C. The resulting supernatant was transferred to a fresh glass vial for analysis. The quality control (QC) sample was prepared by mixing an equal aliquot of the supernatants from all of the samples.

### Metabolomics Analysis

LC-MS/MS analysis was performed using an UHPLC system (Vanquish, Thermo Fisher Scientific) with a UPLC BEH Amide column (2.1 mm × 100 mm, 1.7 μm) coupled to Q Exactive HFX mass spectrometer (Orbitrap MS, Thermo) by Shanghai Biotree Biomedical Technology Co., Ltd., China. The mobile phase consisted of 25 mmol/l ammonium acetate and 25 ammonia hydroxide in water (pH = 9.75) (A) and acetonitrile (B). The auto-sampler temperature was 4°C, and the injection volume was 3 μl.

The QE HFX mass spectrometer was used for its ability to acquire MS/MS spectra on information-dependent acquisition (IDA) mode in the control of the acquisition software (Xcalibur, Thermo). In this mode, the acquisition software continuously evaluated the full-scan MS spectrum. The ESI source conditions were set as follows: sheath gas flow rate at 30 Arb, Aux gas flow rate at 25 Arb, capillary temperature 350°C, full MS resolution at 60,000, MS/MS resolution at 7,500, collision energy at 10/30/60 in NCE mode, and spray voltage at 3.6 kV (positive) or −3.2 kV (negative), respectively.

### Data Processing

The raw data was converted to the mzXML format using ProteoWizard and processed with an in-house program, which was developed using R and based on XCMS, for peak detection, extraction, alignment, and integration. Then, an in-house MS2 database (BiotreeDB) was applied in metabolite annotation. The cutoff for annotation was set at 0.3.

Then, we performed principal component analysis (PCA) and partial least squares discriminant analysis (PLS-DA) by using SIMCA version 16.0.2 (Umetrics AB, Sweden) to obtain an overview of metabolomics data. The contribution of each metabolite was calculated according to the PLS-DA model and expressed as variable importance in the prediction (VIP) score. In order to evaluate the significance of metabolites, the metabolites with a VIP score >1 were analyzed by Student’s *t*-test. The categories of metabolites were defined by using the Human Metabolome Database (HMDB) (https://hmdb.ca/).

### Bioinformatics Analysis

Volcano plots were made using GraphPad Prism V.7.0.0. Meanwhile, we calculated the Euclidean distance matrix for the quantitative value of differential metabolites and clustered the differential metabolites by complete linkage method. Then, we mapped authoritative metabolite databases such as KEGG and PubChem through differential metabolites. After obtaining the matching information of differential metabolites, we searched the pathway database of the corresponding species *Homo sapiens* (human) and conducted an enrichment analysis and a topological analysis to find the most critical pathways that are most related to differential metabolites.

### Receiver Operating Characteristic Curve Analysis

To identify potential diagnostic biomarkers, a receiver operating characteristic (ROC) curve analysis was used to assess the diagnostic potential of differential metabolites, and the area under the curve (AUC) was calculated.

### Statistical Analysis

SPSS 22.0 was used to analyze the data. The results were expressed as mean ± standard deviation (SD) of continuous variables. The normality was tested by Shapiro–Wilk test. Student’s *t*-test, ANOVA, Fisher’s exact test, and Pearson chi square test were used. A *p* value <0.05 was considered statistically significant.

## Results

### Clinical Characteristics of Participants

To investigate the metabolic profile of aqueous humor in RVO-ME, we enrolled 27 age- and sex-matched controls and 28 RVO-ME patients (11 mRVO-ME, 300 μm ≤ CRT < 400 μm, 17 sRVO-ME, CRT ≥ 400 μm) for untargeted metabolomics analysis. There was no significant difference in age, gender, hypertension, coronary heart disease, and diabetes mellitus among the groups (Table 1).

**TABLE 1:** Demographic and clinical characteristics of participants.

	RVO-ME (28)		Control (27)	*p* value^a^
	mRVO-ME (11)	sRVO-ME (17)		
Gender (male/female)	5/6	8/9	12/15	0.986
Age (years), median	70 ± 8.35	70.12 ± 8.03	70.33 ± 8.06	0.992
BMI (kg/m^2^)	23.2 ± 1.73	23.3 ± 1.82	22.88 ± 2.03	0.753
Hypertension (yes/no)	3/8	13/4	19/8	0.907
Diabetes (yes/no)	2/9	3/14	4/23	0.956
Coronary heart disease (yes/no)	2/9	3/14	5/22	0.997
Hyperlipidemia (yes/no)	4/7	6/11	9/18	0.981

RVO-ME, retinal vein occlusion macular edema; mRVO-ME, mild retinal vein occlusion macular edema; sRVO-ME, severe retinal vein occlusion macular edema.

a *P*-value was calculated by Student’s *t*-test.

### AH Metabolism Analysis

**FIGURE 2:**
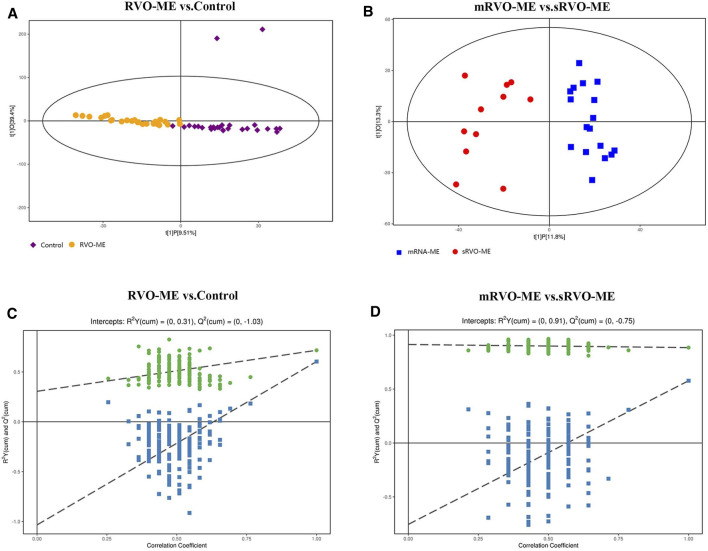
Orthogonal projection to latent structures-discriminant analysis of aqueous humor metabolomic data.

In order to identify the metabolism of aqueous humor, untargeted metabolomics analysis was applied. The results showed that the method had good reproducibility, and only slight changes of the spectral peaks of the QC samples were found (Supplementary Figure S1). A total of 4,945 signals were identified by peak alignment, missing value reconstruction, and data normalization. After Pareto scaling the data, PCA models displayed that QC samples were closely clustered (Supplementary Figure S2), which also indicated the high repeatability of the method and the reliability of the data. In order to visualize and identify the most prominent metabolic differences among the various groups, PLS-DA was performed. Using median coordinates for the training sets, the scatter plot of the latent variables of the PLS-DA models showed good discrimination for comparisons between RVO-ME versus controls and mRVO-ME versus sRVO-ME (Figure 2).

### Differentially Expressed Metabolites Between Groups

**FIGURE 3:**
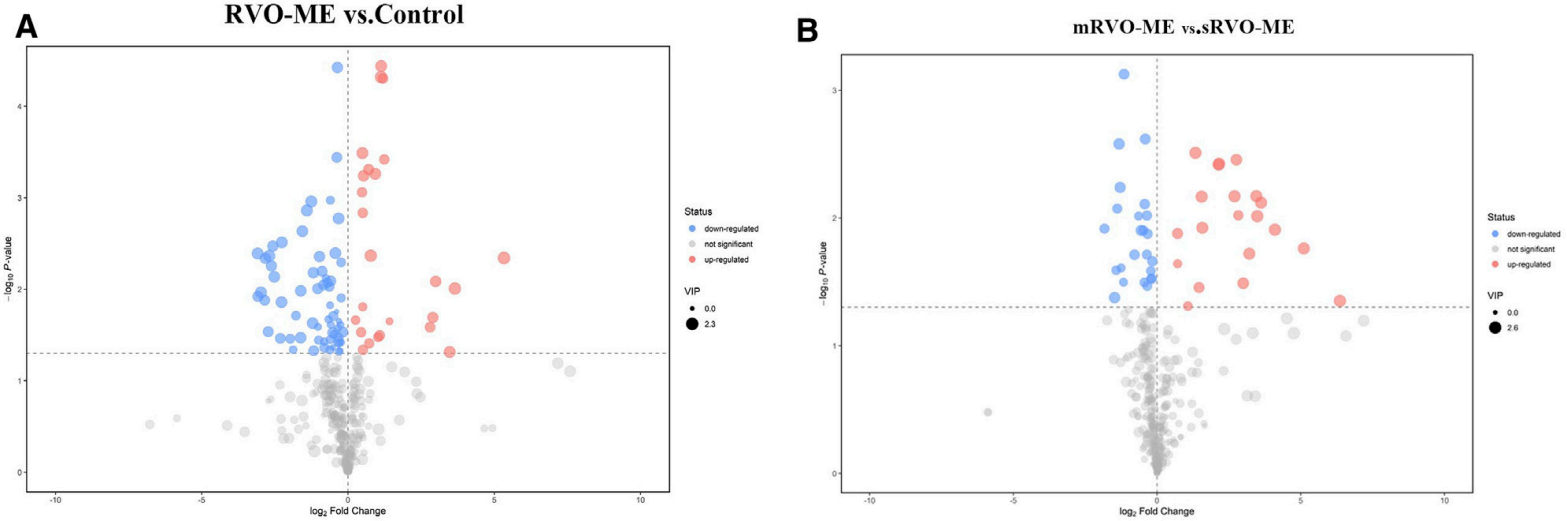
Volcano plots of aqueous humor metabolomic.

**FIGURE 4:**
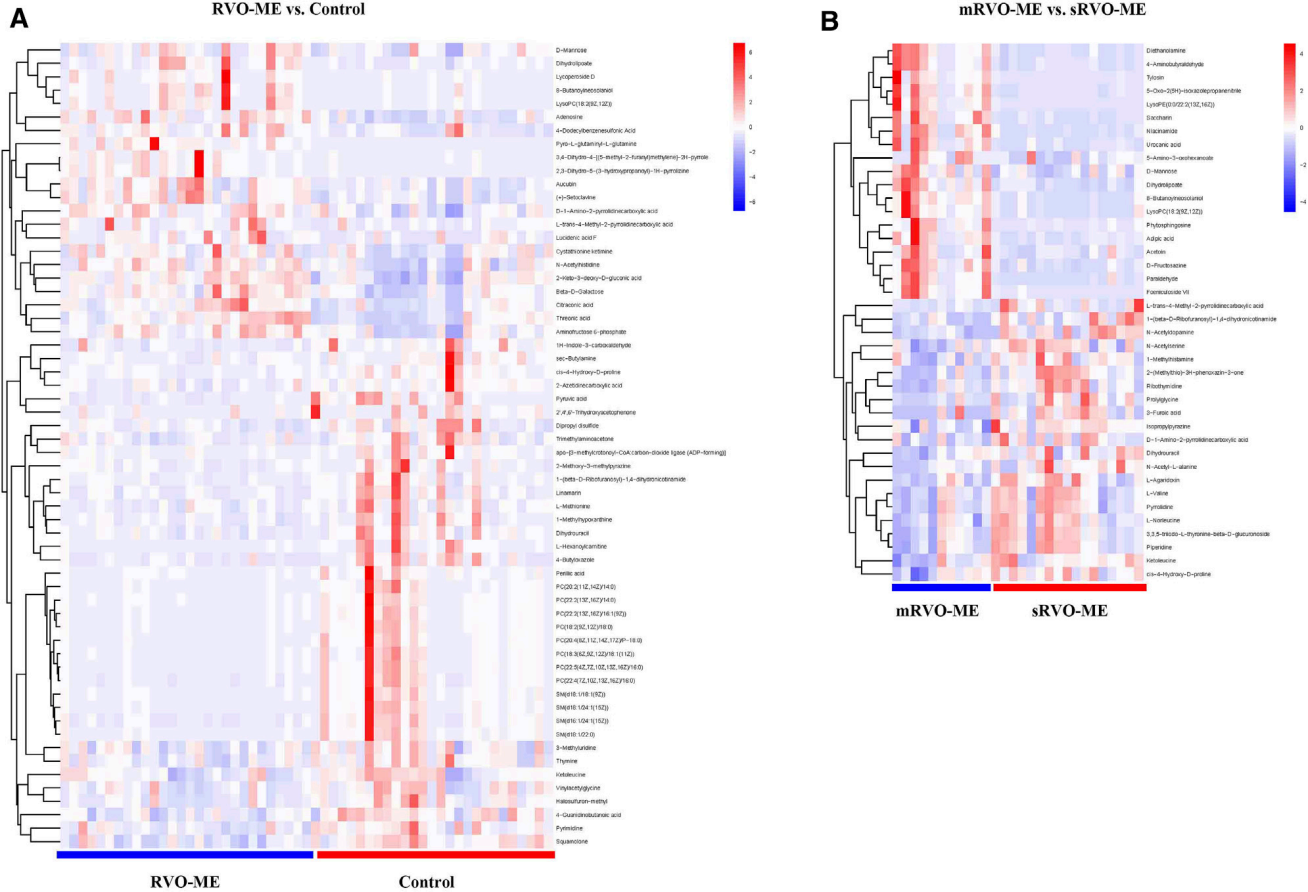
Heat plot of the significantly differential metabolites in RVO-ME.

A total of 60 differential metabolites were found in RVO-ME when compared with controls and 40 differential metabolites in mRVO-ME compared with sRVO-ME (VIP > 1 and *p* < 0.05), including amino acids, carboxylic acids, fatty acid purine, pyrimidine, and so on (Table 2). Volcano plots (Figure 3), heat plot, and hierarchical cluster analysis (Figure 4) were used to investigate variation tendencies for the differential metabolites. Twenty-two metabolites were significantly elevated and 38 metabolites were significantly decreased in RVO-ME compared to controls. We also found 30 increased metabolites and 20 decreased metabolites when comparing mRVO-ME with sRVO-ME.

**TABLE 2:** Identified differential metabolites.

Metabolites	RVO-ME vs. control			mRVO-ME vs. sRVO-ME			Category
	VIP	FC	*p* value	VIP	FC	*p* value^a^	
Ketoleucine	1.158	0.80	0.034	1.86	0.68	0.012	Amino acids
*Cis*-4-Hydroxy-d-proline	1.71	0.70	0.030	1.43	0.89	0.022	Amino acids
8-Butanoylneosolaniol	1.66	7.98	0.008	2.38	10.97	0.007	Fatty acid
Dihydrouracil	1.87	0.74	0.004	1.47	0.88	0.030	Pyrimidones
L-trans-4-methyl-2-pyrrolidinecarboxylic acid	1.06	2.04	0.033	1.06	2.04	0.033	Amino acids
D-mannose	1.17	1.36	0.030	1.17	1.36	0.030	Carbohydrate
Dihydrolipoate	2.18	1.72	0.004	2.48	2.52	0.003	Fatty acids
1-(beta-D-ribofuranosyl)-1,4-dihydronicotinamide	1.51	0.49	0.010	1.70	0.75	0.002	Carbohydrate
D-1-amino-2-pyrrolidinecarboxylic acid	1.18	1.39	0.001	1.15	0.79	0.034	Amino acids
Sec-butylamine	1.15	0.57	0.043	/	/	/	Monoalkyl amines
Adenosine	1.92	2.20	0.000	/	/	/	Purine
Aucubin	1.22	2.36	0.000	/	/	/	Iridoid o-glycosides
Pyruvic acid	2.01	0.38	0.001	/	/	/	Amino acids
1-Methylhypoxanthine	1.70	0.44	0.007	/	/	/	Purine
4-Dodecylbenzenesulfonic acid	1.12	1.65	0.039	/	/	/	Benzenesulfonic acids
2-Keto-3-deoxy-D-gluconic acid	1.98	1.41	0.000	/	/	/	Amino acids
4-Guanidinobutanoic acid	1.37	0.77	0.000	/	/	/	Amino acids
Pyro-L-glutaminyl-l-glutamine	2.33	40.20	0.005	/	/	/	Amino acids
Dipropyl disulfide	1.49	0.54	0.006	/	/	/	Dialkyldisulfides
N-Acetylhistidine	1.57	1.64	0.000	/	/	/	Histidine
3-Methyluridine	1.43	0.88	0.029	/	/	/	Pyrimidine
Thymine	1.88	0.80	0.002	/	/	/	Pyrimidine
Pyrimidine	1.23	0.61	0.008	/	/	/	Pyrimidine
(+)-Setoclavine	1.38	2.29	0.000	/	/	/	Clavines
l-Methionine	1.47	0.56	0.009	/	/	/	Amino acids
1H-indole-3-carboxaldehyde	1.43	0.66	0.008	/	/	/	Indoles
Citraconic acid	1.94	1.90	0.001	/	/	/	Fatty acids
3,4-Dihydro-4-[(5-methyl-2-furanyl)methylene]-2H-pyrrole	1.33	6.99	0.026	/	/	/	Heteroaromatic
PC(22:2 (13Z,16Z)/16:1 (9Z))	1.91	0.13	0.011	/	/	/	Cholines
Threonic acid	2.22	2.18	0.000	/			Sugar acids
Trimethylaminoacetone	1.07	0.71	0.020	/	/	/	Amino acids
Squamolone	1.61	0.78	0.000	/	/	/	Pyrrolidine carboxamides
SM(d18:1/18:1 (9Z))	1.80	0.17	0.007	/	/	/	Phosphosphingolipids
PC[22:5 (4Z,7Z,10Z,13Z,16Z)/16:0]	1.98	0.12	0.004	/	/	/	Phosphatidylcholines
2,3-Dihydro-5-(3-hydroxypropanoyl)-1H-pyrrolizine	1.58	7.47	0.020	/	/	/	Pyrrolizines
SM(d18:1/24:1 (15Z))	1.70	0.14	0.005	/	/	/	Phosphatidylcholines
PC(22:4 (7Z,10Z,13Z,16Z)/16:0)	1.63	0.17	0.003	/	/	/	Phosphatidylcholines
Cystathionine ketimine	1.23	1.43	0.046	/	/	/	Amino acids
Beta-d-galactose	1.73	1.45	0.001	/	/	/	Hexoses
l-Hexanoylcarnitine	1.72	0.33	0.010	/	/	/	Carnitines
apo-[(3-methylcrotonoyl-CoA:carbon-dioxide ligase (ADP-forming)]	1.73	0.33	0.034	/	/	/	Carboximidic acids
Vinylacetylglycine	1.16	0.64	0.009	/	/	/	Amino acids
2-Methoxy-3-methylpyrazine	1.78	0.43	0.024	/	/	/	Methoxypyrazines
PC[18:3 (6Z,9Z,12Z)/18:1 (11Z)]	1.62	0.16	0.006	/	/	/	Cholines
4-Butyloxazole	2.01	0.42	0.001	/	/	/	Oxazoles
PC(20:4 (8Z,11Z,14Z,17Z)/P-18:0)	1.87	0.21	0.014	/	/	/	Cholines
Perillic acid	1.31	0.20	0.034	/	/	/	Menthane monoterpenoids
PC(18:2 (9Z,12Z)/18:0)	1.43	0.14	0.013	/	/	/	Phosphatidylcholines
PC(20:2 (11Z,14Z)/14:0)	1.54	0.12	0.012	/	/	/	Phosphatidylcholines
PC(22:2 (13Z,16Z)/14:0)	1.47	0.15	0.029	/	/	/	Phosphatidylcholines
Linamarin	1.62	0.51	0.004	/	/	/	Cyanogenic glycosides
Lycoperoside	1.94	11.14	0.049	/	/	/	Steroidal saponins
SM(d16:1/24:1 (15Z))	1.84	0.21	0.003	/	/	/	Cholines
Halosulfuron-methyl	1.87	0.34	0.002	/	/	/	Carboxylic acids
SM(d18:1/22:0)	2.08	0.15	0.004	/	/	/	Cholines
Lucidenic acid F	1.31	2.12	0.032	/	/	/	Triterpenoids
Aminofructose 6-phosphate	1.24	1.42	0.001	/	/	/	Triterpenoids
LysoPC(18:2 (9Z,12Z))	2.21	12.57	0.010	/	/	/	Phosphocholines
2′,4′,6′-Trihydroxyacetophenone	1.35	0.44	0.047	/	/	/	Alkyl-phenylketones
l-Norleucine	/	/	/	1.26	0.78	0.019	Amino acids
3,3,5-triiodo-l-thyronine-beta-D-glucuronoside	/	/	/	1.44	0.78	0.010	Steroid glucuronide conjugates
l-Valine	/	/	/	1.14	0.86	0.026	Amino acid
Niacinamide	/	/	/	2.16	6.78	0.003	Nicotinamide
Foeniculoside VII	/	/	/	2.56	17.16	0.012	Terpene glycosides
Piperidine	/	/	/	1.38	0.80	0.013	Piperidines
Urocanic acid	/	/	/	1.39	7.10	0.010	Carboxylic acids
Prolylglycine	/	/	/	1.29	0.28	0.012	Dipeptides
Diethanolamine	/	/	/	2.58	4.47	0.004	1,2-Aminoalcohols
Isopropylpyrazine	/	/	/	1.51	0.74	0.008	Pyrazines
Phytosphingosine	/	/	/	2.14	7.99	0.032	1,3-Aminoalcohols
Saccharin	/	/	/	2.41	12.29	0.008	Benzothiazoles
8-Butanoylneosolaniol	/	/	/	2.38	10.97	0.007	Fatty acid
5-Oxo-2(5H)-isoxazolepropanenitrile	/	/	/	2.54	11.22	0.010	Isoxazoles
Pyrrolidine	/	/	/	1.13	0.87	0.030	Amino acids
D-Fructosazine	/	/	/	2.42	4.42	0.004	Pyrazines
Paraldehyde	/	/	/	2.49	6.48	0.007	Trioxanes
1-(beta-D-ribofuranosyl)-1,4-dihydronicotinamide	/	/	/	1.70	0.75	0.002	Glycosylamines
5-Amino-3-oxohexanoate	/	/	/	1.07	2.10	0.049	Medium-chain keto acids
D-1-amino-2-pyrrolidinecarboxylic acid	/	/	/	1.15	0.79	0.034	Amino acids
2-(Methylthio)-3H-phenoxazin-3-one	/	/	/	2.00	0.40	0.003	Phenoxazines
Ribothymidine	/	/	/	1.87	0.41	0.006	Pyrimidine nucleosides
Adipic acid	/	/	/	2.42	2.99	0.012	Fatty acids
L-Agaridoxin	/	/	/	1.51	0.58	0.019	Amino acids
N-Acetyl-l-alanine	/	/	/	1.27	0.38	0.008	Amino acids
4-Aminobutyraldehyde	/	/	/	2.49	2.93	0.007	Alpha-hydrogen aldehydes
N-Acetylserine	/	/	/	1.38	0.72	0.013	Amino acids
3-Furoic acid	/	/	/	2.03	0.36	0.042	Furoic acids
1-Methylhistamine	/	/	/	1.10	0.74	0.032	2-Arylethylamines
Acetoin	/	/	/	1.74	2.75	0.035	Acyloins
N-acetyldopamine	/	/	/	1.66	0.45	0.001	Catechols
LysoPE (0:0/22:2 (13Z,16Z))	/	/	/	2.39	34.45	0.017	Phosphoethanolamines
LysoPC(18:2 (9Z,12Z))	/	/	/	2.43	9.26	0.019	Phosphocholines
Tylosin	/	/	/	2.55	82.48	0.045	Aminoglycosides

RVO-ME, retinal vein occlusion macular edema; mRVO-ME, mild retinal vein occlusion macular edema; sRVO-ME, severe retinal vein occlusion macular edema; VIP, variable importance in the projection; FC, fold change.

a *P-value* was calculated by Student’s *t*-test.

### Pathway Analysis of Differential Aqueous Metabolites

**FIGURE 5:**
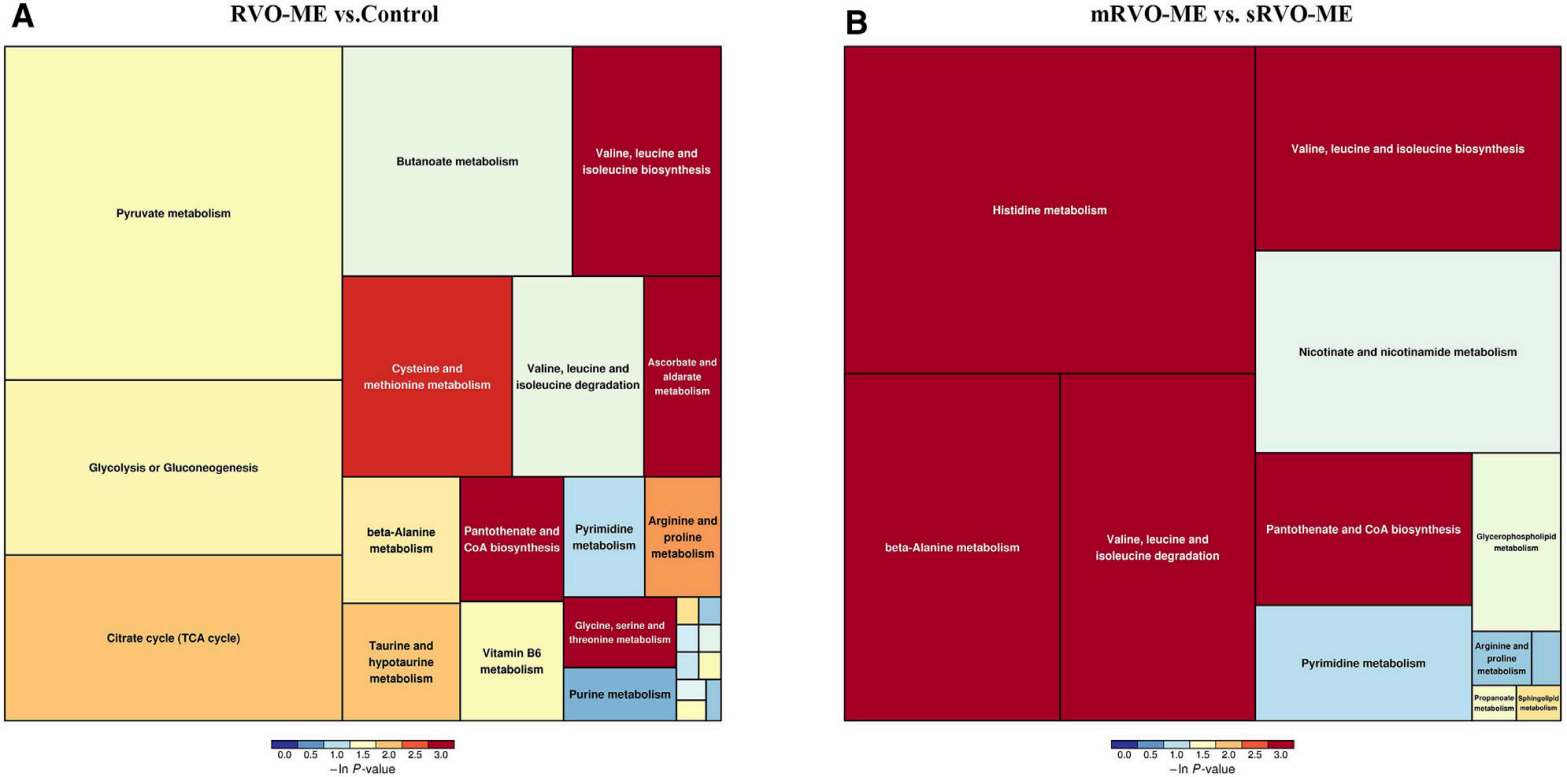
Pathway analysis of differential aqueous metabolites in each group.

MetaboAnalyst was applied to compare metabolic disturbances in RVO-ME versus controls and mRVO-ME versus sRVO-ME (Table 3). When comparing RVO-ME patients with controls, a total of three differential pathways were found, namely, valine, leucine, and isoleucine biosynthesis; pantothenate and coenzyme A (CoA) biosynthesis; and ascorbate and aldarate metabolism. Valine, leucine, and isoleucine biosynthesis; pantothenate and CoA biosynthesis; beta-alanine metabolism; histidine metabolism; and valine, leucine, and isoleucine degradation were altered in sRVO-ME when compared to mRVO-ME (Figure 5).

**TABLE 3:** The significantly altered pathways in RVO-ME.

Pathway	RVO-ME vs. controls		sRVO-ME vs. mRVO-ME	
	*p* value^a^	Metabolites	*p* value^a^	Metanolites
Valine, leucine, and isoleucine biosynthesis	<0.001	Pyruvic acid; citraconic acid; 4-methyl-2-oxopentanoate	0.016	l-valine; 4-methyl-2-oxopentanoate
Pantothenate and CoA biosynthesis	0.016	Dihydrouracil; pyruvic acid	0.016	Dihydrouracil; l-valine
Ascorbate and aldarate metabolism	0.043	Pyruvic acid; threonic acid	–	–
Beta-alanine metabolism	–	–	0.018	4-Aminobutyraldehyde; dihydrouracil
Valine, leucine, and isoleucine degradation	–	–	0.035	l-valine; M-4-methyl-2-oxopentanoate
Histidine metabolism	–	–	0.042	Urocanic acid; 1-methylhistamine

RVO-ME, retinal vein occlusion macular edema; mRVO-ME, mild retinal vein occlusion macular edema; sRVO-ME, severe retinal vein occlusion macular edema.

a *P-value* was calculated by Student’s *t*-test.

### ROC Curve Analysis

**FIGURE 6:**
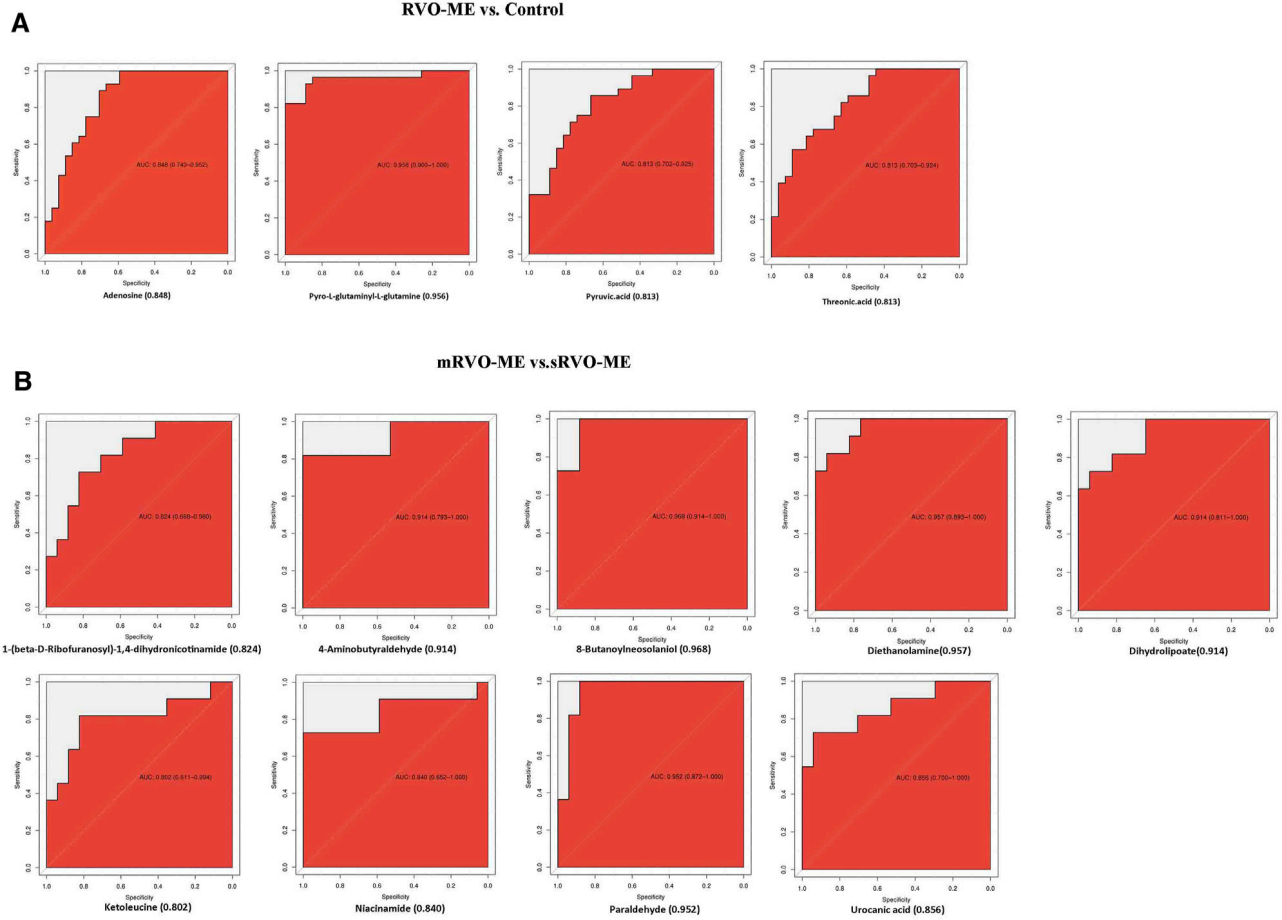
Receiver operating characteristic curve analysis.

Further screening of the metabolic indicators was conducted by ROC analysis (Figure 6). As shown in Figure 6A, threonic acid, pyro-L-glutaminyl-l-glutamine, adenosine, and pyruvic acid had an AUC ≥ 0.813 for distinguishing RVO-ME from controls. When comparing sRVO-ME with mRVO-ME patients, the ROC analysis showed that nine metabolites had an AUC ≥ 0.848, including urocanic acid, 1-(beta-D-ribofuranosyl)-1,4-dihydronicotinamide, phytosphingosine, niacinamide, 8-butanoylneosolaniol, dihydrolipoate, paraldehyde, 4-aminobutyraldehyde, and diethanolamine (Figure 6B).

## Discussion

In the present study, we explored the metabolomic changes in AH of patients with RVO-ME disease. To the best of our knowledge, this is the first time that UHPC-Q-TOF/MS was used to analyze the discrepancy of AH metabolomics in RVO-ME versus controls and mRVO-ME versus sRVO-ME. After correction, 60 and 40 metabolites were differentially expressed in RVO-ME versus controls and mRVO-ME versus sRVO-ME, respectively. Notably, amino acids were the most abundant differential metabolite category. Also, significant alterations were noted in several metabolic pathways. Interestingly, we found that pantothenate and CoA biosynthesis and valine, leucine, and isoleucine biosynthesis were significantly altered both in RVO-ME versus controls and mRVO-ME versus sRVO-ME. Additionally, ROC curves were also performed to assess the metabolites of AH, which could best distinguish RVO-ME from the controls and mRVO-ME from sRVO-ME.

Previous studies have revealed that intraocular angiogenic factors and inflammatory cytokines play pivotal roles in the occurrence and progression of ocular complications in patients with RVO (An et al., 2021; Yong et al., 1007). In the current study, many inflammation-related metabolites have also been found in RVO-ME when compared with controls. Adenosine is an endogenous purine nucleoside, which is widely distributed in the body and interacts with G-protein-coupled receptors (Sebastião and Ribeiro, 2009; Santiago et al., 2020). Under the stress conditions of tissue ischemia, hypoxia, and inflammatory response, the concentration of extracellular adenosine increased exponentially. Previous studies have shown that adenosine or its analogues could raise intraocular angiogenic factors and inflammatory cytokines, such as vascular endothelial growth factor, insulin-like growth factor-1, basic fibroblast growth factor, interleukin-8, and angiogenin-2 (Feoktistov et al., 2003; Haskó and Pacher, 2012). Luo et al. (2019) found that adenosine attenuated the inflammatory response of human endothelial cells through negative regulation of Toll-like receptor MyD88 signal. Haas et al. (2011) showed that adenosine could induce a reduction of Toll-like receptor4 expression at the surface of human macrophages, resulting in a robust inhibition of TNF-α production. In this study, compared with the controls, the level of adenosine in RVO-ME increased, which suggests that RVO-ME was associated with inflammatory process, to some extent. In addition, adenosine also regulates vascular tension and thus blood flow. Adenosine, acting predominantly at A_2A_R, induced the production of NO, which causes vasodilation of retinal vessels (Riis-Vestergaard et al., 2014; Riis-Vestergaard and Bek, 2015). We speculated that the decrease of A_2A_R also resulted in the increase of free adenosine, which might play a crucial role in RVO vascular occlusion. Threonic acid, also known as threonate, is a central signaling hub in ascorbate–aldarate pathway (Wang et al., 2019). We detected an abnormal expression of threonine and ascorbate and aldarate metabolism in RVO-ME. As shown by the experiment of corneal neovascularization in a rodent model, ascorbic acid might inhibit angiogenesis, which was regarded as a vital event of RVO (Ashino et al., 2003). In our present study, we also found that adenosine and threonic acid could act as a potential biomarker to distinguish RVO-ME from the controls according to ROC analysis. Therefore, we hold the view that adenosine and threonic acid may play a crucial role in RVO-ME.

When compared with mRVO-ME, we found that the level of D-mannose decreased in sRVO-ME. D-mannose is a natural C-2 epimer of glucose, which can be transported to mammalian cells through the plasma membrane to promote the diffusion of glucose transporter (GLUT). Rehak et al. (2010) found that IL-1 was rapidly and strongly up-regulated in the retina and retinal pigment epithelium (to levels 80 times higher than controls) in RVO-ME, whereas D-mannose can inhibit macrophage IL-1 (Torretta et al., 2020) and delay the development of osteoarthritis *in vivo* by enhancing autophagy activated by the AMPK pathway (Lin et al., 2021). Consequently, we considered that D-mannose supplementation may be a meaningful treatment for macular edema caused by RVO.

Oxidative stress played a momentous role in the occurrence and prognosis of RVO-ME (Chen et al., 2018; Hwang et al., 2020); accordingly, related metabolic abnormalities were also found in our study. Many amino acids expressed differently in our study were also involved in oxidative stress response, such as glycine (Knebel et al., 2012), histidine (Nasri et al., 2020), methionine (Demerchi et al., 2021), N-acetylserine (Kim et al., 2019), urocanic acid (Jauhonen et al., 2011), cis-4-hydroxy-D-proline (Aswani et al., 2019), et al. We also detected a significant metabolite in nucleotide metabolism: nicotinamide (NAM). NAM, the amide of vitamin B3 and precursor for nicotinamide adenine dinucleotide (NAD+), has a strong antioxidant property and can effectively reduce the damage to cells caused by reactive oxygen species (ROS) during oxidative stress (Mejía et al., 2017). Compared with mRVO-ME, the level of NAM in sRVO-ME was decreased. In addition, the AUC of NAM was found to be 0.848 in the ROC analysis, which could serve as a potential biomarker for differentiation between sRVO-ME and mRVO-ME. We believe that the degree and prognosis of RVO-ME are closely related to oxidative stress response. NAM may become an important prognostic biomarker for the treatment of RVO-ME.

Another interesting finding from our study was that the pantothenate (PA) and CoA biosynthesis and valine, leucine, and isoleucine biosynthesis pathways showed a difference in mRVO-ME versus sRVO-ME and RVO-ME versus controls, respectively. Therefore, we speculated that these two metabolic pathways were not only correlated to the occurrence of RVO-ME but also affected the severity of RVO macular edema. Studies had reported that these two metabolic pathways were associated to oxidative stress. PA can regulate cell membrane CoA synthesis and protect endothelial function from enhanced oxidative stress (Demirci et al., 2014). Many studies had also confirmed that this metabolic pathway was abnormal in a variety of diseases, like diabetic kidney disease (Tao et al., 2020), neurodegeneration (Zizioli et al., 2015), Vogt–Koyanagi–Harada (Xu et al., 2021), et al. Valine, leucine, and isoleucine, namely, branched-chain amino acids (BCAAs), which could over-induce oxidative processes and up-regulate proinflammatory factors (Zhenyukh et al., 2017), are unable to be synthesized by animals. Hence, a variety of pathological changes, such as maple syrup urine disease (MSUD) (Xu et al., 2020), type 2 diabetes (Zeng et al., 2019), and cancer (Peng et al., 2020; Sivanand and Vander Heiden, 2020) could be detected when there is a disorder in BCAA metabolism. Nevertheless, the molecular mechanisms of BCAAs involved in the pathogenesis of RVO-ME-inducing retinopathy remain unknown, thus warranting future research.

We recognized the limitations of our research. Firstly, the sample size of patients was small, due to the difficulty in collecting AH sample from patients and controls. Secondly, obtaining AH samples for the diagnosis of RVO-ME or judgment of severity and prognosis of macular edema is not so practical to perform due to the invasive nature of the procedure. Subsequent studies will recruit more participants and combine serum or urine sample analysis to strengthen the results. Thirdly, owing to geographical limitations, our research was limited to the Chinese Han population. We look forward to get the results of other ethnic samples from other researchers. Last but not the least, further research is needed to shed more light on the exact role of these metabolites and relevant metabolic pathways in the pathogenesis of RVO-ME.

In conclusion, to our knowledge, this study is the first one to provide a comprehensive understanding of the metabolomics of AH in patients with RVO-ME. The results showed that a series of complex and serious metabolic disorders occurred in AH in patients with RVO-ME. Furthermore, we also found significant differences in metabolites between mild macular edema and severe macular edema in RVO-ME patients. Significantly, intraocular angiogenic factors, inflammatory mechanisms, and oxidative stress response may play a prominent role in the occurrence and development of RVO-ME. The above-mentioned results may elucidate the metabolic biomarkers for the prognosis and novel therapeutic strategies to prevent or delay the development of RVO-ME.

## Data Availability

The datasets presented in this study can be found in online repositories. The names of the repository/repositories and accession number(s) can be found in the article/Supplementary Material.
