# Supplementary figures and images for: Metabolite Changes in the Aqueous Humor of Patients With Retinal Vein Occlusion Macular Edema: A Metabolomics Analysis
Source: Front Cell Dev Biol. 2021 Dec 21;9:762500. doi: 10.3389/fcell.2021.762500 (PMC8724431; doi:10.3389/fcell.2021.762500)

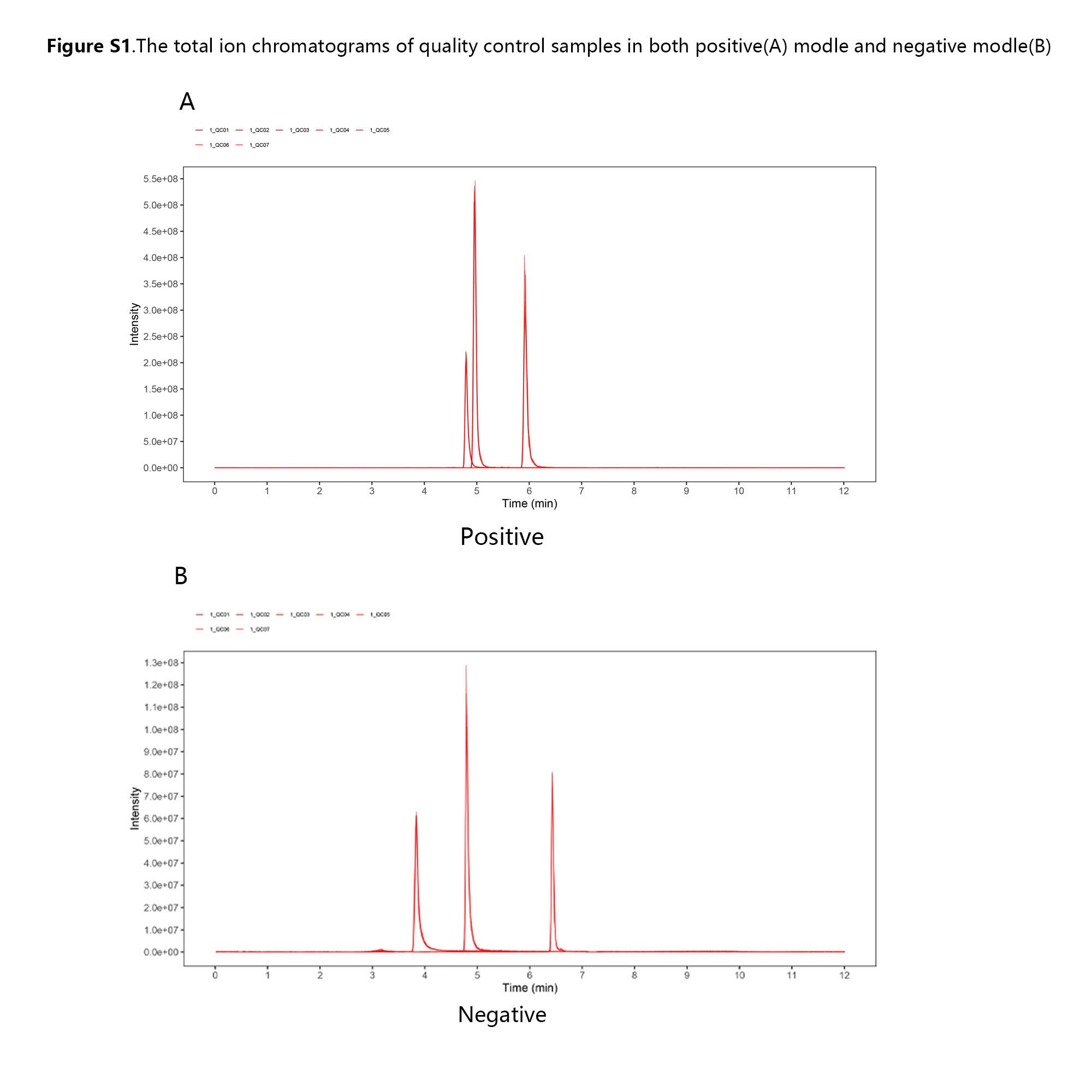

Supplement: Supplementary file 1 [file Image1.JPEG]

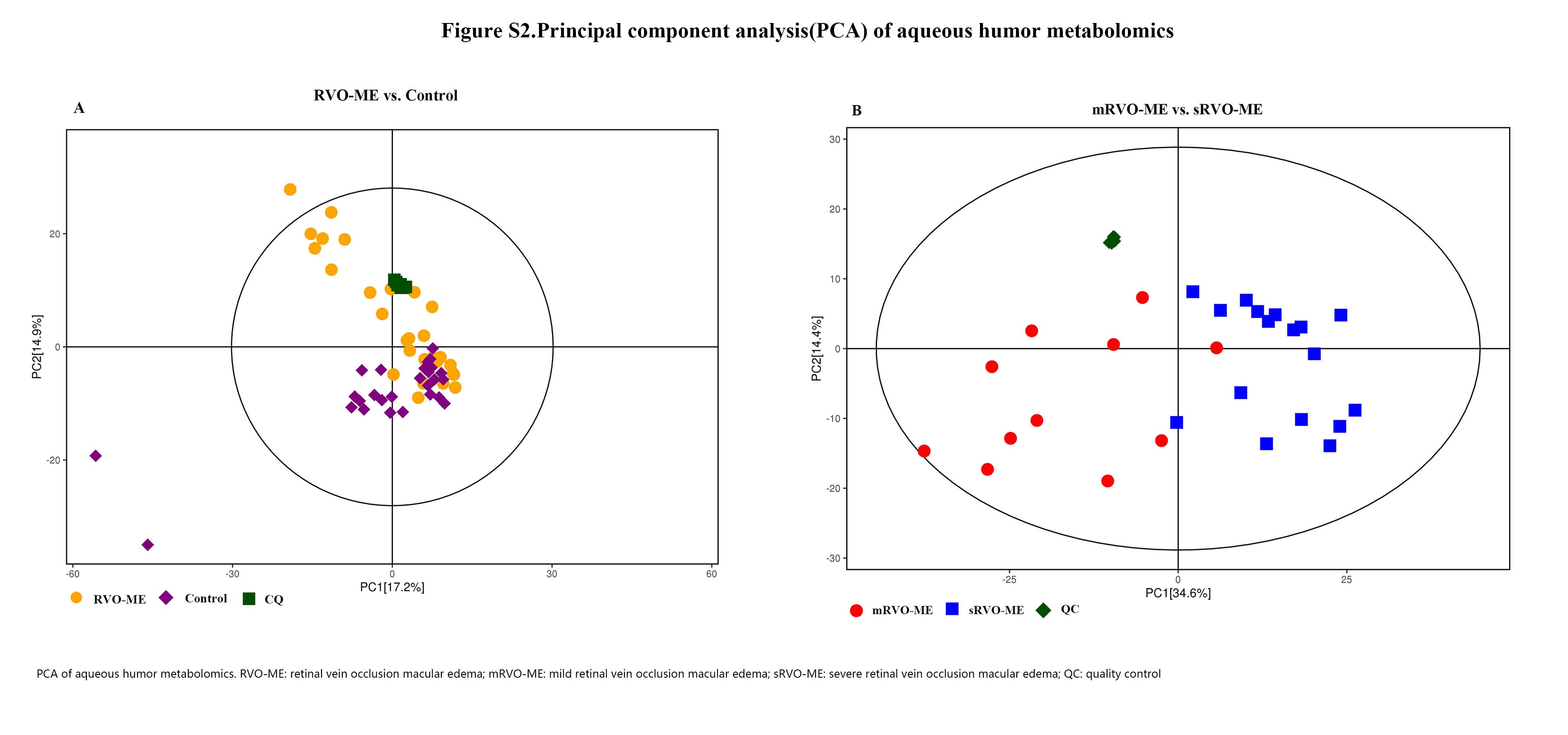

Supplement: Supplementary file 2 [file Image2.JPEG]
